# Breast density classification using frequency-based features in microwave imaging

**DOI:** 10.1038/s41598-025-28629-8

**Published:** 2025-11-27

**Authors:** Mehran Taghipour-Gorjikolaie, Banafsheh Khalesi, Bilal Khalid, Navid Ghavami, Mario Badia, Mohammad Ghavami, Gianluigi Tiberi

**Affiliations:** 1 School of Engineering, London South Bank University, London, UK; 2 UBT - Umbria Bioengineering Technologies, Perugia, Italy

**Keywords:** Breast Cancer, Breast Density, Classification, Frequency-based Features, Machine Learning, Microwave Imaging, Cancer, Engineering, Health care, Oncology

## Abstract

Breast cancer remains one of the leading causes of death among women worldwide. One major challenge in early and accurate detection is breast density. High breast density not only obscures tumors on current imaging modalities, making them harder to identify, but also significantly increases the likelihood of diagnostic errors, both by medical professionals and automated detection systems. As a result, accurately classifying the breast density is crucial, and can lead to better, more tailored screening approaches and reduce the chances of error. This is especially critical for younger women, who are usually excluded from national screenings due to concerns such as radiation exposure. Microwave imaging offers a promising solution to this problem. Unlike traditional imaging methods, it uses safe, non-ionizing radiation, making it suitable for women of all ages. Beyond its safety, microwave imaging has the potential not only to detect breast cancer, but also to classify breasts into high or low density. This dual capability allows for more personalized and accurate cancer detection based on breast density, improving outcomes and reducing diagnostic uncertainty. Our microwave imaging prototype called MammoWave works by scanning the breast using a wide range of low-power electromagnetic signals captured from multiple positions around the breast. This approach provides a rich set of data that helps create an internal map of breast tissue without exposing patients to harmful radiation. This technique makes it possible to extract frequency-based characteristics from both the spatial and spectral domains, taking advantage of not just the signal’s magnitude but also its phase information. These rich features can offer deeper insights into tissue composition and improve the accuracy of breast density classification. Our analysis shows that by fusing features from both the magnitude and phase of the signals—and focusing on approximately the first 40 components of the fast Fourier transform (FFT)—it’s possible to achieve an accuracy of around 70% in classifying breast density using a support vector machine (SVM) with a radial basis function (RBF) kernel. Furthermore, instead of using the full frequency range (1 to 9 GHz), selecting specific sub-bands (1, 3, 4, 5, and 6 GHz) can improve the accuracy to approximately 73%. Importantly, the results also reveal that when breast density is correctly identified and taken into account, the performance of machine learning models in detecting breast cancer improves significantly boosting specificity and sensitivity by around 10% and 5%, respectively for low-density breasts, and by 15% and 10% respectively for high-density breasts.

## Introduction and related works

Tissue density is a fundamental physical property that plays a critical role in medical imaging and diagnosis. It refers to the amount of mass per unit volume of a tissue and influences how structures appear in radiographic modalities such as X-ray, ultrasound, and magnetic resonance imaging (MRI). For instance, bone, being highly calcified, appears radiopaque (white) on X-rays due to its high density, while softer tissues like fat and muscle display in varying shades of gray based on their relatively lower density^1^. Among soft tissues, the human breast exhibits substantial interindividual variability in tissue density, which carries important implications for both breast cancer risk and the effectiveness of detection strategies. The breast is primarily composed of fibroglandular (dense) and adipose (fatty) tissue. The term breast density (BD) refers to the proportion of fibroglandular tissue relative to fatty tissue as seen on a mammogram. This characteristic is not only a physical descriptor but also a key factor influencing breast cancer risk and the effectiveness of imaging-based detection methods^2^.

Moreover, BD significantly affects breast cancer detection by decreasing mammographic sensitivity and increasing the likelihood of missed diagnoses. Numerous recent studies have shown that as BD increases, the sensitivity of standard mammography decreases dramatically; sensitivity can drop from around 75% to just over 50%^3^ contributing to a higher incidence of interval cancers, rising from approximately 1.8 to 7.9 cases per 1,000 women^4,5^. A recent UK trial demonstrated that supplemental imaging in women with extremely dense breasts, such as contrast-enhanced mammography or abbreviated MRI, detected up to 3.5 times more cancers than mammography alone, with the potential to save hundreds of lives annually^6^. These findings highlight the critical role of BD assessment in improving early detection, optimizing screening pathways and reducing missed diagnoses^3^.

According to the American College of Radiology’s BI-RADS (breast imaging reporting and data system), BD is classified into four categories: (A) almost entirely fatty, (B) scattered areas of fibroglandular density, (C) heterogeneously dense, and (D) extremely dense. Women in the latter two categories are considered to have dense breasts, a condition that affects approximately 40–50% of women undergoing screening mammography^1,7^. One of the big challenges in developing machine learning models with these four groups is lack of enough data in some groups, which could make the classification less robust. Therefore, to obtain balanced classes suitable for machine learning analysis, grouping BI-RADS A and B as low density (LD) and BI-RADS C and D as high density (HD) is considered. This binary grouping is widely used in breast imaging studies and retains clinical significance. Clinically, BD is significant in two major respects. First, high BD is an independent risk factor for breast cancer; women with extremely dense breasts are four to six times more likely to develop breast cancer than those with fatty breasts^2,8^. Second, it poses a serious diagnostic challenge: dense fibroglandular tissue and malignant tumours both appear radiopaque on mammograms, reducing lesion visibility and increasing false-negative rates^9^. This so-called “masking effect” contributes to delayed diagnoses and higher rates of interval cancers, particularly in dense breast populations^10^.

In response to these diagnostic limitations, various supplemental imaging modalities have been introduced, including digital breast tomosynthesis (DBT), ultrasound, and MRI. These techniques offer improvements such as enhanced soft tissue contrast and three-dimensional imaging. However, some—such as MRI—can be costly, resource-intensive, or involve ionizing radiation (in the case of DBT), which may limit their use for routine or frequent screening^11^.

Over the past decade, image processing techniques, model-based algorithms, and advanced computational methods such as machine learning (ML) and deep learning (DL) have been widely adopted to automate BD classification and support breast cancer risk assessment. These approaches have been applied across various imaging modalities, including mammography, MRI, and ultrasound. DL has shown notable success with mammography and MRI, largely due to the availability of extensive annotated datasets and the inherent richness of these image types. While mammography remains the standard for BD evaluation, MRI is increasingly valued for its superior soft-tissue contrast. These characteristics make both modalities well suited for DL-based and algorithm-driven computational analysis.

For example, the study of Payne et al.^3^ employed non–DL automated tools such as Volpara, a physics-based software that estimates volumetric BD from digital mammograms by analyzing X-ray attenuation, breast thickness, and tissue composition, to systematically evaluate the impact of BD on detection sensitivity. Also, in Jing et al.^12^, the authors proposed a hybrid approach that combines a convolutional neural network (CNN) for automatic breast tissue segmentation with radiomic feature extraction and ML classification. This method significantly reduced inter-observer variability—an issue common in manual MRI interpretation—highlighting the potential of automated techniques to enhance consistency and reliability in clinical workflows. Similarly, the study of Bezek et al.^13^ introduced a quantitative ultrasound-based method for BD estimation by measuring the speed of sound through tissue using conventional ultrasound transducers, leveraging the fact that dense tissue conducts sound faster than fatty tissue.

In addition, numerous studies have applied ML and DL models to classify BD from mammographic images, aiming to enhance the accuracy of breast cancer detection. For example, the study of Saffari et al.^14^ demonstrated the effectiveness of combining a CNN with a conditional generative adversarial network (cGAN) for accurate BD classification from mammograms. A range of other machine learning approaches have also been explored: the study of Liu et al.^15^ employed a directed acyclic graph support vector machine (DAG-SVM); Muštra et al.^16^ utilized the k-nearest neighbors (k-NN) algorithm; and studies by Liu et al., Gong et al., and Sansone et al.^17–19^ implemented SVM models. In Arefan et al.^20^, a feedforward artificial neural network (ANN) with a single hidden layer was used for classification. The authors in^21^ proposed a hybrid hierarchical framework that integrated principal component analysis (PCA) for feature reduction with multiple classifiers—including k-NN, probabilistic neural network (PNN), ANN, naïve fuzzy classifier (NFC), and SVM. Additionally, Dhou et al.^22^ introduced a DL-based method incorporating the U-Net architecture for BD segmentation and classification.

Collectively, these studies illustrate the evolution of ML in this domain, from traditional algorithms like SVM, k-NN, and random forest (RF) to more sophisticated DL architectures such as CNNs and U-Net. This progression reflects an ongoing effort to improve classification accuracy, reduce inter-observer variability, and enhance clinical outcomes in breast cancer screening. However, a common limitation among these approaches is their reliance on mammographic data, which is inherently affected by ionizing radiation and reduced sensitivity in women with dense breasts.

To overcome these challenges, there is a growing interest in alternative, non-ionizing imaging techniques, particularly microwave imaging (MWI). MWI differentiates tissues based on their dielectric properties (variations in permittivity and conductivity), rather than radiographic opacity. Malignant breast tumours typically exhibit elevated water content compared to surrounding tissues, offering a distinct contrast mechanism in the microwave spectrum^23^. MWI is generally categorized into microwave tomography, which reconstructs dielectric property maps, and radar-based imaging, which uses reflected signals to detect anomalies. The non-ionizing, low-cost, and portable nature of MWI makes it particularly well-suited for frequent and safe screening, including among younger women and high-risk populations. Despite promising results in recent studies applying ML for classifying tumours and reconstructing images from MWI data^24,25^ the impact of BD—particularly its classification—on the performance of microwave-based detection systems remains largely unexplored. Prior work has examined microwave-based routes for estimating or stratifying breast density across radar imaging, handheld radio-frequency metrology, and transmission imaging. In Iriarte et al.^26^ the authors have reported discrimination of lucent versus dense breasts on non-symptomatic scans via MARIA^®^ radar platform. A handheld calliper (Mi Scan, 3–8 GHz) has shown good agreement with mammographic BI-RADS and high binary non-dense/dense accuracy without compression^27^. Transmission microwave imaging has linked reconstructed permittivity statistics to mammographic density in healthy cohorts, with significant between-group separation and SVM-based categorisation using average-permittivity, thresholding, k-means, and pixel-level metrics^28^. Complementary clinical work with a two-antenna system (SAFE) has demonstrated density-stratified lesion-classification performance, underscoring the value of modelling density within microwave workflows^29^. Together, these studies indicate that microwave measurements encode composition cues suitable for density assessment. Unlike image reconstruction or device-specific pipelines, we classify LD/HD directly from calibrated complex S-parameters by deriving dual-domain (angular–spectral) fast Fourier transform (FFT) features from magnitude and phase over selected sub-bands and training an SVM-RBF classifier—a reconstruction-free, signal-level design that, to our knowledge, is novel for breast-density estimation. This reconstruction-free, signal-level formulation extends microwave signal classification practice and places our contribution precisely within—and beyond—the existing literature.

The primary goal of this research was to develop a robust and reliable ML model capable of classifying BD into two main categories: high density and low density. A secondary objective was to investigate whether BD influences the sensitivity and specificity of microwave-based detection systems, and to assess if incorporating density information into ML models can enhance diagnostic performance. Unlike mammography, where dense breast tissue often obscures tumours, MWI operates on electromagnetic rather than radiographic principles and may be less affected by high-density tissue. The variation in data collected from the MWI device, represented in the complex domain, enables the application of frequency-based techniques for extracting features across both spatial and spectral domains.

The rest of the manuscript is organized as follows: the next section describes the feature extraction methodology in detail. Subsequently, we present the experimental results, including an ablation study on the proposed frequency-based method, the performance of various classifiers, and the effect of combining signals from different frequency sub-bands. This section also discusses the impact of BD on cancer detection. Finally, the last section offers conclusions and an in-depth discussion of the findings.

## Proposed method

### Dataset

This research has been done based on data collected from two MammoWave clinical trials (Clinicaltrials.gov identifiers NCT04253366 and NCT05300464) carried out at four EU Hospitals. All volunteers underwent MammoWave examination in addition to the conventional breast examination path, which was used as reference standard. Conventional breast examination path included mammogram; thus, data related to the mammographic breast density was collected. In more details, each mammogram (either full-field digital mammography or DBT) was independently double-read by two radiologists, with consensus or arbitration for any discordant interpretations. According to the reference standard, all scanned breasts were classified into LD and HD breasts. In total, 1197 samples were collected, of which 582 cases were LD and 615 HD. Table 1 summarizes the information about collected data. Also, conventional breast examination path allowed to classify the 1197 samples in: 187 samples having histology-confirmed tumour (thus, labelled as non-healthy); 1,010 samples having no lesion or benign lesions (thus, labelled as healthy). Clinical data used in this investigation were collected in the context of MammoWave clinical trials, approved by the Ethics Committee of CEAS Umbria, Italy (24197/22/AV, 09/03/2020) which acts as coordinator. The study was carried out in accordance with the protocol and principles of Declaration of Helsinki and the guidelines of Good Clinical Practice issued by ICH. All subjects included in the study gave informed consent to participate.

**Table 1:** Summary of collected data from 2020 to 2023.

Hospital	Year	Number of patients	Number of samples	Age range	Reference standard, i.e. the radiologic study output	
					Low density (LD)	High density (HD)
Hospital 1	2020	180	352	20–78	177	175
	2023	106	203	18–77	87	116
Hospital 2	2020	110	217	19–78	130	87
	2023	130	252	27–79	118	134
Hospital 3	2020	63	125	22–77	53	72
Hospital 4	2023	24	48	31–87	17	31
**Total**	–	613	1197	18–87	582	615

**Fig. 1:**
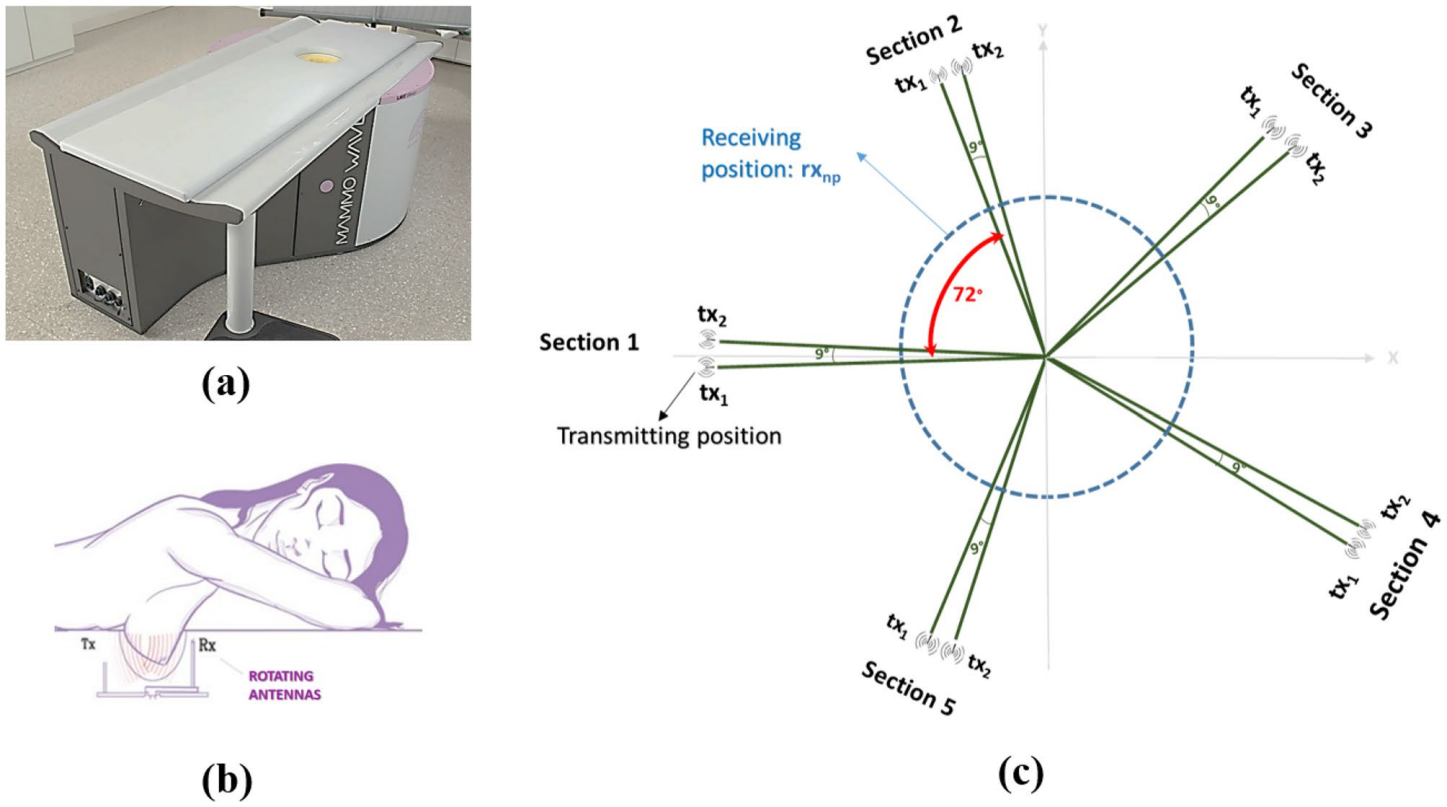
(**a**) MammoWave prototype, (**b**) sketch of MammoWave’s scanning configuration, (**c**) transmitting and receiving antenna configurations.

Before starting to explain the proposed method, it is necessary to be familiar with the structure of the MammoWave device, and the idea why we decided to use frequency-based analysis to extract features for BD classification. MammoWave device is equipped with two antennas (a transmitter (Tx) and a receiver (Rx)) which work in frequency range of 1 to 9 GHz (5 MHz sampling) and rotate around the medium (breast) for a 360$^\circ$ scan. As shown in Fig. 1, the positions of the transmitter around a breast are $[0^\circ ,9^\circ ,72^\circ ,81^\circ ,\dots ,297^\circ ]$ equalling 10 positions, while the receiver scans every $4.5^\circ$ around the breast resulting in 80 positions. In each pair position of the transmitter and receiver, S21 complex parameters are collected via a vector network analyser (VNA), resulting in an $800\times 1601$ matrix $M\in \mathbb {C}^{800\times 1601}$. As Equation (1) describes, each 80 rows of the matrix correspond to 80 positions of the receiver and one position of the transmitter^30^. This means that we can restructure the matrix into ten $80\times 1601$ matrices.

1 $$\begin{aligned} M \;\longrightarrow \;\{M^{(t)} \in \mathbb {C}^{R\times F} \mid t=1,2,3,\dots ,T\} \end{aligned}$$

where, $R$, $T$, and $F$ are the number of receiving positions, transmitting positions, and frequency points, respectively. And $M^{(t)}$ is the sub-matrix corresponding to the $t$th transmitter position and all associated receiver positions.

Assessment of the variations of collected complex S21 parameters can show important spatial and spectral patterns. This assessment can be done in two different ways, across different dimensions of the S21 matrix. Let us consider a sub-matrix (80 × 1601) containing information about a Tx and 80 positions of Rx. Each column relates to a fixed frequency and describes how electromagnetic response is changing across different positions of the receiver. This spatial pattern reflects how signals propagate through tissues and interact with varying internal structures. On the other hand, assessing this variation across the rows of the matrix provides an RF-spectral profile of the transmission characteristics at a fixed receiver position, which means how tissue properties affect signal transmission over frequency.

### Frequency-based feature extraction

**Fig. 2:**
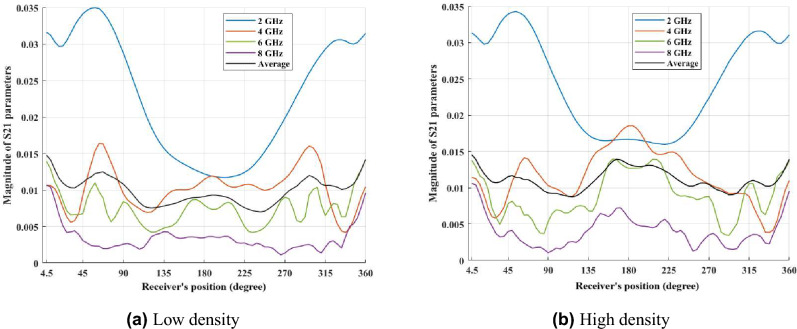
Average spatial patterns (magnitude) at selected frequencies. Each plot shows the mean magnitude of S21 across receiver positions for a fixed frequency (2, 4, 6, 8 GHz), averaged across multiple representative breasts in each density class (LD, HD). Averages are shown to emphasise typical class behaviour (individual breasts vary). The spatial traces illustrate that HD breasts tend to display richer oscillatory structure across receiver angle whereas LD breasts appear smoother in the averaged response. This difference is confirmed through a quantitative analysis of standard deviation and the coefficient of variation.

**Fig. 3:**
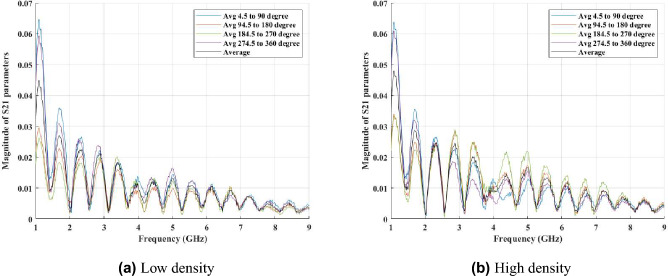
Average RF-spectral profiles (magnitude) at representative receiver positions. Each panel shows the mean spectral response (S21 magnitude versus frequency) at selected receiver positions, averaged across multiple representative breasts in each class. The averaged spectra highlight class differences: LD tends to show a smoother spectral decay while HD exhibits more frequency-dependent structure. These plots motivate the use of frequency-domain features in the classification pipeline.

The main goal of this paper is to classify the density of the breast; therefore, it would be useful to see the spatial and RF-spectral characteristics in different real cases. To do this, we randomly selected 40 healthy breasts with high and low density. After providing an average matrix for each case among sub-matrices $M^{(t)}$, for each density class an average matrix is generated among all selected samples. **Spatial patterns** are obtained by fixing a frequency and observing how S21 varies across the 80 receiver positions for a single transmitter angle; these patterns characterise how the transmitted wavefront is modulated around the breast and therefore reflect large-scale scattering and angular heterogeneity. **RF-spectral patterns** are obtained by fixing a receiver position and observing how S21 varies across frequency; these profiles characterise frequency-dependent permittivity and absorption effects. Figures 2 and 3 present averages computed across multiple representative breasts from each density class. We use averaged plots intentionally: averaging reduces case-by-case noise and highlights the typical class behaviour. In these averaged views we observe that, for the same measurement geometry, HD breasts typically show richer spatial oscillations across receiver angle and more pronounced frequency-dependent structure, while LD breasts tend to exhibit smoother spatial variation and a more gradual spectral decay. To clarify this observation, we measured the standard deviation and the coefficient of variation (CV) of the S21 magnitude across receiver positions. For instance, at 4 GHz, the standard deviation was approximately 5.73 $\times$ 10$^{-5}$ for HD and 4.66 $\times$ 10$^{-5}$ for LD, indicating about 23% higher spatial variability in dense breasts. The CV values were 0.46 for HD and 0.53 for LD. At 6 GHz, the standard deviation increased by about 14% in HD compared with LD, with corresponding CV values of 0.62 and 0.68. These findings confirm that HD breasts generate slightly stronger spatial fluctuations due to greater internal scattering and tissue heterogeneity, while LD breasts display smoother and more uniform responses. Such quantitative and qualitative differences motivated the use of our dual-domain FFT approach and the extraction of both magnitude and phase features from spatial and RF-spectral transforms.

These patterns reveal the possibilities of extracting frequency-based features. FFT is a powerful tool for converting spatial or temporal data into the frequency domain, allowing us to capture oscillation patterns, quantify signal complexity, and extract spectral signatures that are not apparent in the raw S21 matrix. FFT across receiver positions can highlight dominant spatial frequencies and quantify tissue heterogeneity, and across frequency points can extract spectral energy distribution, entropy or spectral flatness, and peak frequency shifts or width.

To extract frequency-based features, the first step is to eliminate the DC (zero frequency) bias by removing the mean value across the frequency axis as shown in Equation (2). It can help to remove receiver-wise average across frequencies, which may include uninformative background signals or system bias.

2 $$\begin{aligned} M_{\textrm{filtered}}^{(t)}[i,j] = M^{(t)}[i,j] \;-\; \frac{1}{F}\sum _{k=1}^{F}M^{(t)}[i,k] \quad \text {for }i=1,\dots ,R,\;j=1,\dots ,F \end{aligned}$$

By using Equations (3) and (4), we can apply Fourier transform and extract FFT coefficients for spatial and RF-spectral patterns, respectively.

3 $$\begin{aligned} & \textrm{FFTC}_{\textrm{RF}\text {-}\textrm{spectral}}[i,k] = \sum _{n=0}^{F-1} S21[i,n]\;e^{-j\,\frac{2\pi }{F}kn},\quad k=0,1,2,\dots ,F-1 \end{aligned}$$

4 $$\begin{aligned} & \textrm{FFTC}_{\textrm{Spatial}}[k,j] = \sum _{n=0}^{R-1} S21[n,j]\;e^{-j\,\frac{2\pi }{R}kn},\quad k=0,1,2,\dots ,R-1 \end{aligned}$$

where, *R* and *F* are maximum number of receivers and frequency, respectively. Since the S21 matrix contains complex values, FFTC are complex. Therefore, we can use Equations (5) and (6) to extract magnitude and phase of these coefficients.

5 $$\begin{aligned} & |X[k]|= \sqrt{\bigl (\Re (X[k])\bigr )^2 + \bigl (\Im (X[k])\bigr )^2} \end{aligned}$$

6 $$\begin{aligned} & \angle X[k] = \arctan \bigl (\Im (X[k]),\Re (X[k])\bigr ) \end{aligned}$$

Magnitude and phase of both RF-spectral and spatial characteristics are then used in equations (7) to (10). By applying FFT on RF-spectral responses, energy distribution across oscillations in different frequencies can be extracted. Magnitude of the FFT shows whether the tissue responds in a smooth or oscillatory way. Also, the phase of FFT captures rapid changes or shifts in frequency, possibly reflecting microstructural differences in tissue density. Therefore, the expectation is that since high-density breasts tend to scatter more, they would cause richer high-frequency content and more complex frequency responses.

7 $$\begin{aligned} & A_{\mathrm {RF\text {-}spectral}}[i,:] = \bigl |\textrm{FFTC}_{\mathrm {RF\text {-}spectral}}[i,:]\bigr | \end{aligned}$$

8 $$\begin{aligned} & \Phi _{\mathrm {RF\text {-}spectral}}[i,:] = \angle \textrm{FFTC}_{\mathrm {RF\text {-}spectral}}[i,:] \end{aligned}$$

Applying FFT on spatial response transfers information into spatial frequencies. The magnitude can show how rapidly the signal oscillates across space, which indicates inhomogeneity or heterogeneity in the internal structure of the breast. Moreover, the phase can reveal spatial phase shift, and extract wavefront distortions due to differing tissue types or boundaries. These spatial-based components are less in low density breasts as they normally show smoother spatial signals.

9 $$\begin{aligned} & A_{\textrm{spatial}}[:,j] = \bigl |\textrm{FFTC}{\textrm{Spatial}}[:,j]\bigr |\end{aligned}$$

10 $$\begin{aligned} & \Phi _{\textrm{spatial}}[:,j] = \angle \textrm{FFTC}{\textrm{Spatial}}[:,j] \end{aligned}$$

Subsequently, statistical features such as mean ($\mu$) and standard deviation ($\sigma$) are used as final extracted features. Moreover, to prevent biasing the final model to magnitude or phase, and to use the advantages of both, Z-score normalization as shown in Equation (11) is applied before concatenating, because this method is not so sensitive to outliers. As shown in Equation (12), all these statistical information from magnitude and phase of FFT from RF-spectral and spatial domains are concatenated to make a feature vector for $M^{(t)}$; finally by concatenating these features from sub-matrices, the final features are generated as shown in Equation (13).

11 $$\begin{aligned} & {\text {X}}_{\mathrm{standardized}} \;=\; \frac{X - \mu }{\sigma } \end{aligned}$$

12 $$\begin{aligned} & {F^{(t)}} = \{\mu _{\textrm{mag}}^{\mathrm {RF\text {-}spectral}}, \sigma _{\textrm{mag}}^{\mathrm {RF\text {-}spectral}}, \mu _{\textrm{phase}}^{\mathrm {RF\text {-}spectral}}, \sigma _{\textrm{phase}}^{\mathrm {RF\text {-}spectral}}, \mu _{\textrm{mag}}^{\textrm{Spatial}}, \sigma _{\textrm{mag}}^{\textrm{Spatial}}, \mu _{\textrm{phase}}^{\textrm{Spatial}}, \sigma _{\textrm{phase}}^{\textrm{Spatial}}\} \end{aligned}$$

13 $$\begin{aligned} & F = \{F^{(1)},F^{(2)},F^{(3)},\dots ,F^{(10)}\} \end{aligned}$$

**Fig. 4:**
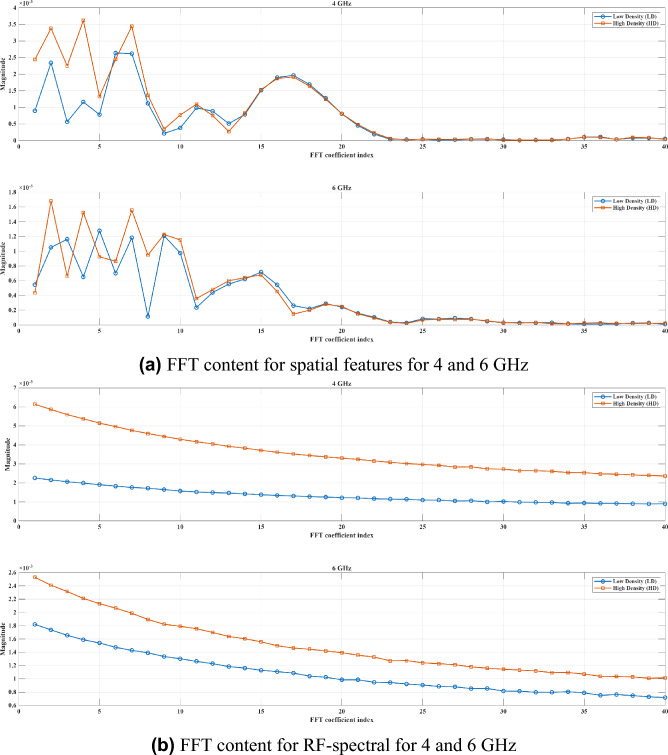
Example comparison of FFT components for frequencies 4 and 6 GHz.

As shown in Fig. 4a and b, the first 40 FFT magnitude components are compared between high- and low-density breasts for the same cases illustrated in Figs. 2 and 3. Figure 4a presents the FFT content of spatial patterns at 4 and 6 GHz, while Fig. 4b shows the FFT content of RF-spectral patterns at the same frequencies.

As it can be seen, different frequency bandwidths include different FFT components that can lead to extract strong features for classification purposes. The selection of frequency sub-bands is motivated by the propagation behaviour of microwaves in biological tissue. Lower frequencies penetrate more deeply into the breast, allowing assessment of global tissue properties, while higher frequencies have shallower penetration but provide greater sensitivity to fine structural variations. Considering both ranges therefore provides complementary information that is useful for distinguishing between density classes.

## Results

### Ablation analysis

To develop robust and reliable models, Monte Carlo and k-fold cross validations have been used. Based on Monte Carlo validation, in every run 20% of the data was randomly selected as test data and the remaining 80% samples were used as training and validation data. Additionally, 4-fold cross-validation was applied within the training data to systematically evaluate and identify the most suitable models. As a result, all reported performance metrics represent the average values across multiple runs, thereby providing a comprehensive assessment of model stability and generalizability. In this section, our objective is to identify more suitable frequency-based features for the classification task. We will then compare the most effective representation of FFT features with other well-known features reported in the literature and used by previously published models. It is worth noting that, to ensure a fair ablation analysis and to maintain focus on identifying the most discriminative features, a k-NN classifier is employed throughout the evaluation. According to the initial assessment k=5 was selected, as it provided a good balance between stability and sensitivity to local structure in the dataset. Using a very small k (such as 1) made the results more sensitive to noise, while larger values of k tended to over smooth the decision boundary.

**Fig. 5:**
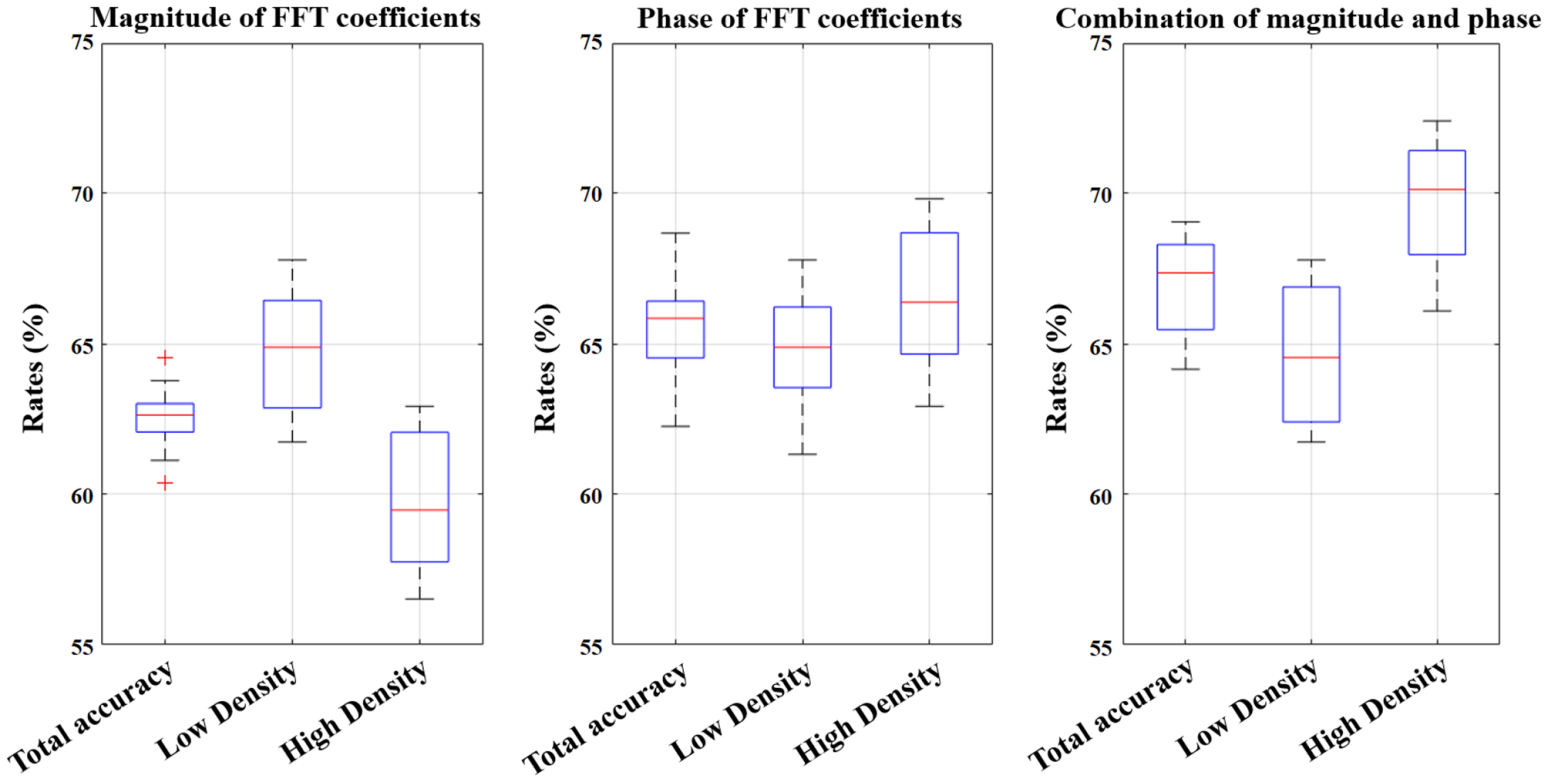
Box chart for magnitude, phase and combination of output feature-based features for verification data.

**Fig. 6:**
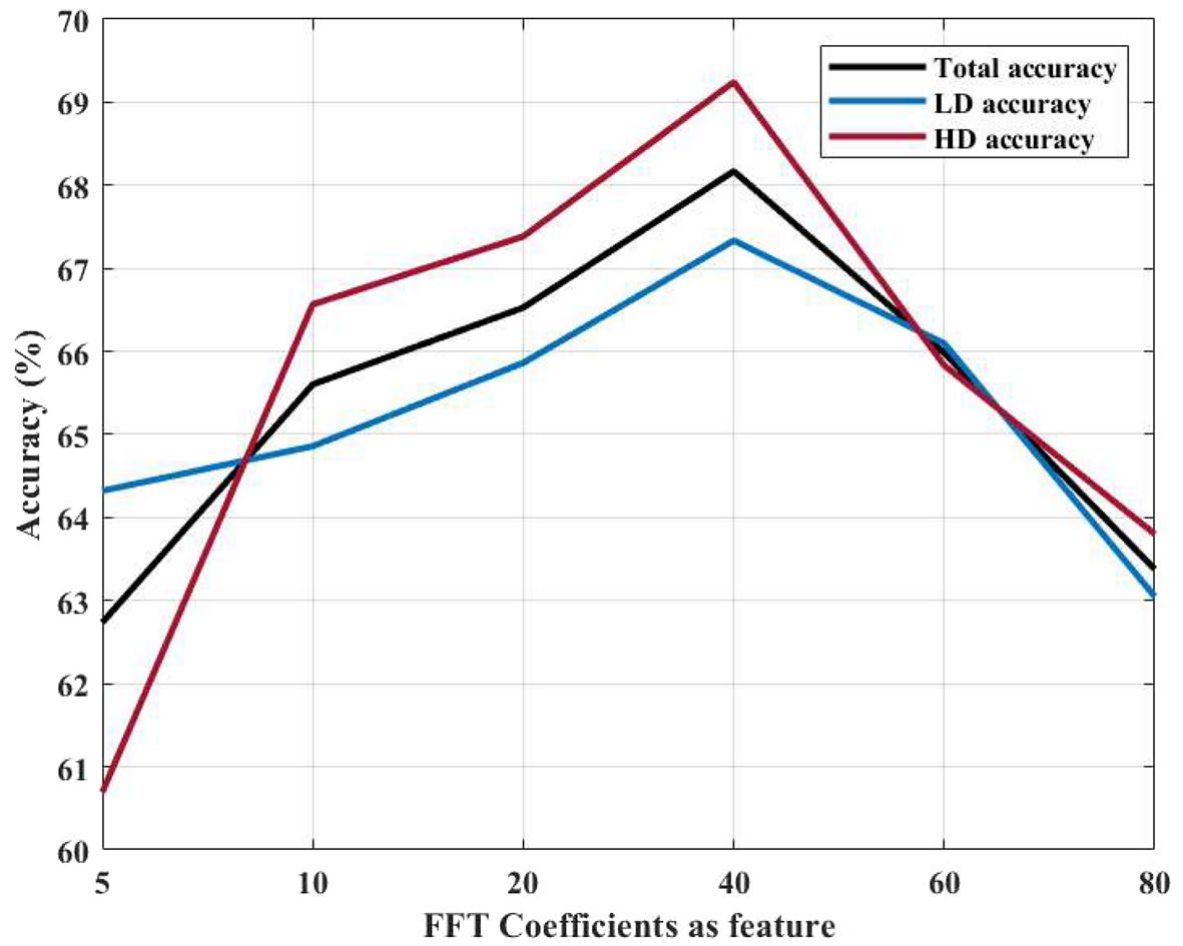
Variation in classification accuracy with respect to the number of FFT coefficients used as features for breast density classification.

Since both the S21 parameters and frequency components of FFT analysis are complex, it is possible to extract statistical features from magnitude, phase or both. Moreover, the number of FFT coefficients (components) can play an important role in classification of density. By considering the first 10 components of FFT analysis, Fig. 5 shows an average result over 5 Monte Carlo and 4-fold cross validation for training and validation data. In addition, as shown in Fig. 6, variation of classification accuracy can be seen for different numbers of components for combination of features. As it can be seen, the classification accuracy stabilises when using around 40 FFT components, which indicates that these coefficients capture the dominant frequency content of the signals while avoiding redundancy and noise from higher-order terms.

### Comparison of different features

**Fig. 7:**
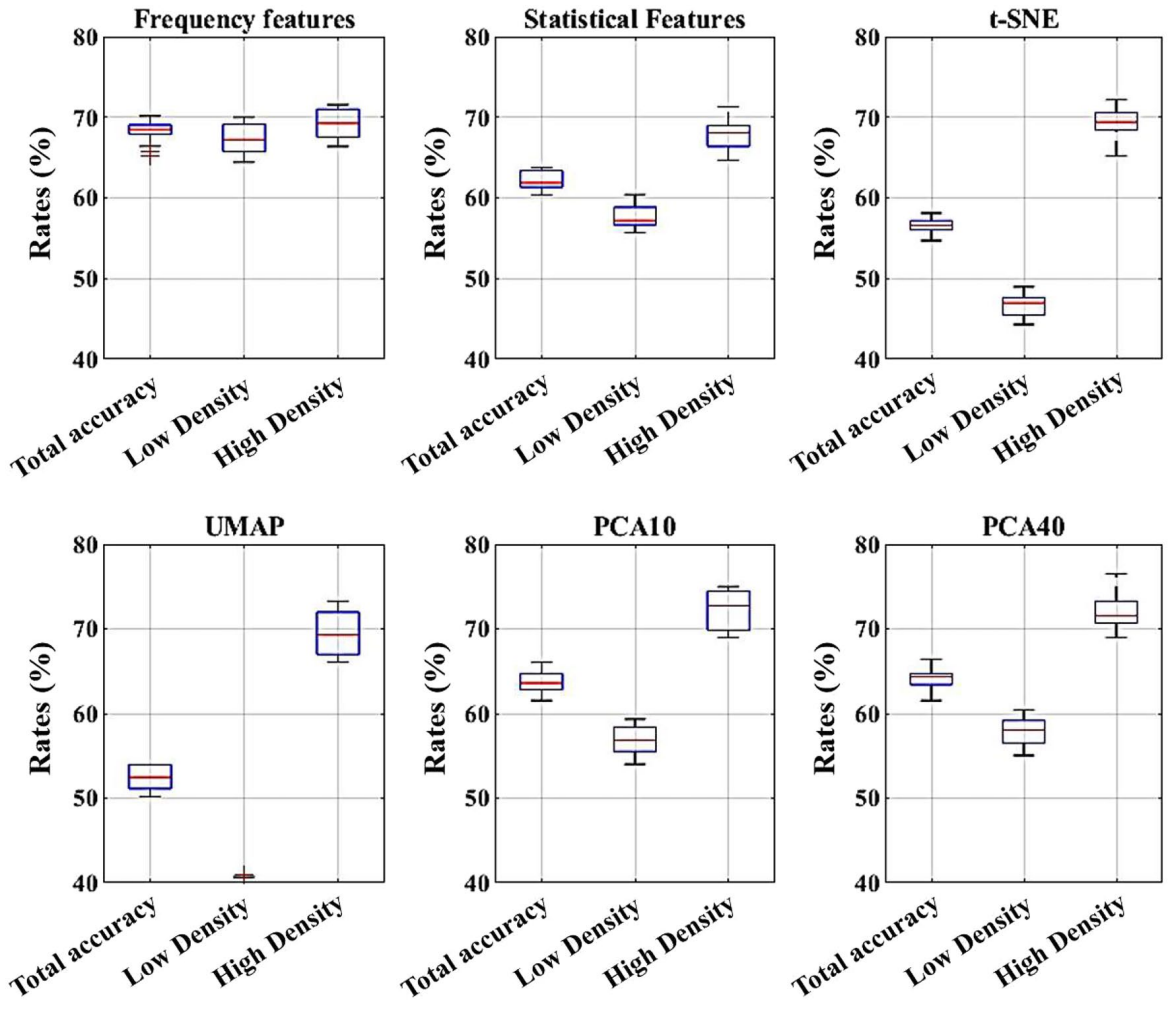
Box chart for different types of features..

In this section, the proposed features are compared with several widely used and well-established feature extraction techniques commonly employed in ML applications, particularly in the context of BD classification. These benchmark methods include statistical features (SF), PCA, t-distributed stochastic neighbor embedding (t-SNE), and uniform manifold approximation and projection (UMAP). Like the proposed method, these comparative feature extraction techniques are applied to each sub-block, corresponding to the different transmitter positions. For SF, the extracted metrics include mean, standard deviation, minimum, maximum, median, range, and Shannon index. In the case of PCA, feature sets consisting of the top 10 and top 40 principal components are considered. For both t-SNE and UMAP, the features are reduced to two dimensions to ensure consistency and comparability across methods. The comparison aims to evaluate the effectiveness of the proposed approach relative to these standard techniques in capturing the most discriminative information for accurate classification. Figure 7 shows median and standard deviations of the validation data for different types of features.

### Comparison of different classifiers

In this section, for each combination we want to see the performance of different well-known classifiers on validation and test dataset to propose the best structure for classification of BD. Evaluated classifiers from the simplest ones to more complicated are: 5-Nearest Neighbour, naive bayes (NB) classifier, SVM-RBF, decision tree, RF, RF ensemble, extreme gradient boosting (XGBoost), adaptive boosting (Adaboost) and also three DL based models; namely deep neural network (DNN), long short-term memory (LSTM) and 1-Dimention CNN (1D-CNN). It is worth noting that the DNN consisted of four fully connected hidden layers with 256, 128, 64, and 32 neurons using ReLU activation, followed by a dropout layer (rate = 0.3) to prevent overfitting. The LSTM network comprised one recurrent layer with 64 memory units, a dense layer with 32 neurons, and a dropout rate of 0.2. The 1D-CNN included two convolutional layers (32 and 64 filters, kernel size = 3) followed by max-pooling and a dense layer with 64 neurons. All models used a sigmoid activation function in the output layer, the Adam optimizer (learning rate = 0.001), binary cross-entropy loss, and were trained for 50–60 epochs with small batch sizes (8–16) to ensure stable convergence.

**Fig. 8:**
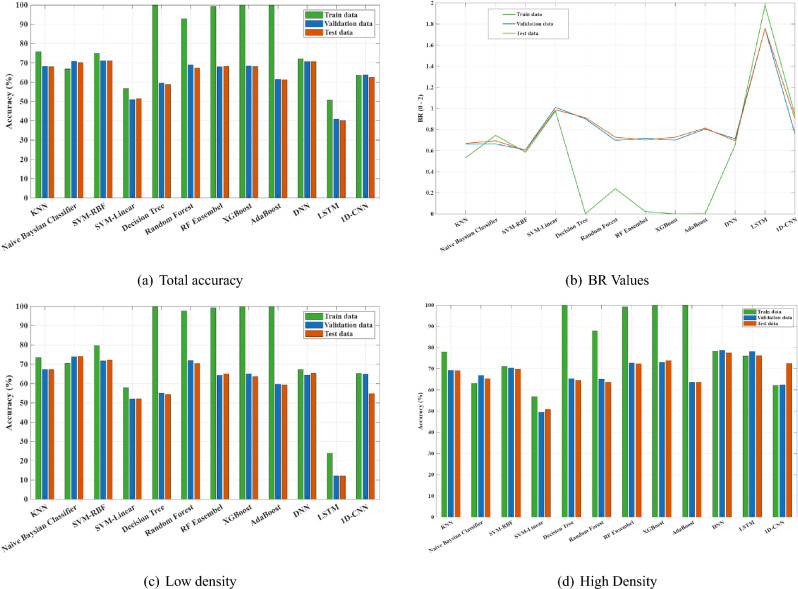
Bar chart for different classifiers.

Figure 8a,c,d represent the accuracy of classification for total, low and high BD classification, respectively. Moreover, balance rate (BR)^30^ as shown in Equation (14) is used to ensure balance accuracy for low and high density. As discussed in^30,31^, Equation (14) defines the BR, which evaluates how evenly a classifier performs on sensitivity and specificity: the first term penalises low overall performance, and the second penalises imbalance between the two. Unlike the F1-score, which includes false positives and false negatives but does not guarantee balance, BR ensures that both sensitivity and specificity contribute fairly, making it more suitable for medical applications. Figure 8b shows the variation of BR among classifiers.

14 $$\begin{aligned} \mathrm {Balance\ Rate\ (BR)} = \bigl (2 - (\textrm{sensitivity} + \textrm{specificity})\bigr ) + \min \bigl (\textrm{sensitivity} - \textrm{specificity}\bigr ) \end{aligned}$$

### Frequency selection

**Fig. 9:**
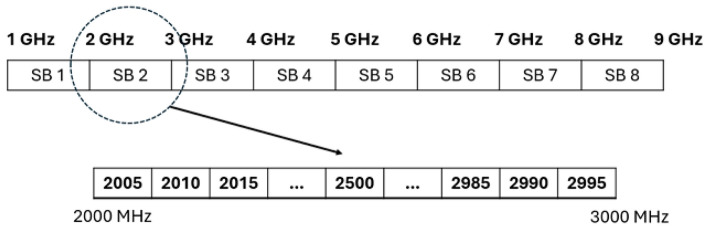
Division of 1–9 GHz frequency range into eight 1 GHz sub-bands (SB1–SB8), for frequency selection analysis^30^.

As illustrated in Fig. 9, the 1–9 GHz frequency range was divided into eight sub-bands (SB1–SB8), each covering approximately 1 GHz. This division is motivated by the observation that within each 1 GHz segment, the spectral patterns of the measured signals remain relatively consistent, without major variations across nearby frequencies. Treating each sub-band as a unit therefore simplifies the analysis while still capturing the essential frequency-dependent behaviour. In this section, the aim is to find the best combination of bandwidths (frequencies) to increase accuracy and robustness. In this part, according to the performance of SVM-RBF, we continued with this classifier to find a more robust, reliable, and accurate model for BD classification. Tables 2 and 3 show the obtained results for different combinations of frequency bandwidths for validation and test data.

**Table 2:** Impact of different bandwidths on breast density classification for validation data.

Bandwidths		Total accuracy (%)	LD accuracy (%)	HD accuracy (%)	BR (0–2)
1	1000 0000	$70.85 \pm 1.023$	$67.40 \pm 1.054$	$75.29 \pm 1.585$	$0.65 \pm 0.029$
2	0100 0000	$67.53 \pm 0.961$	$63.28 \pm 0.983$	$73.00 \pm 1.531$	$0.73 \pm 0.287$
3	0010 0000	$68.25 \pm 2.092$	$63.85 \pm 2.292$	$73.91 \pm 2.539$	$0.72 \pm 0.046$
4	0001 0000	$67.30 \pm 1.188$	$62.88 \pm 1.246$	$73.00 \pm 1.733$	$0.74 \pm 0.032$
5	0000 1000	$67.98 \pm 1.429$	$62.92 \pm 1.525$	$74.52 \pm 1.947$	$0.74 \pm 0.036$
6	0000 0100	$65.28 \pm 2.161$	$60.03 \pm 2.373$	$72.06 \pm 2.600$	$0.80 \pm 0.047$
7	0000 0010	$66.21 \pm 1.558$	$61.84 \pm 1.675$	$71.83 \pm 2.063$	$0.76 \pm 0.038$
8	0000 0001	$66.96 \pm 1.262$	$62.38 \pm 1.331$	$72.87 \pm 1.798$	$0.75 \pm 0.033$
1, 3, 5, 7	1010 1010	$69.96 \pm 0.849$	$68.64 \pm 0.853$	$71.66 \pm 1.431$	$0.63 \pm 0.027$
2, 4, 6, 8	0101 0101	$69.58 \pm 1.704$	$67.30 \pm 1.843$	$72.53 \pm 2.192$	$0.65 \pm 0.040$
3, 4, 5, 6	0011 1100	$70.57 \pm 2.035$	$68.17 \pm 2.226$	$73.65 \pm 2.487$	$0.64 \pm 0.045$
1, 2	1100 0000	$70.30 \pm 0.808$	$67.40 \pm 0.806$	$74.04 \pm 1.394$	$0.65 \pm 0.026$
7, 8	0000 0011	$66.38 \pm 1.766$	$61.91 \pm 1.915$	$72.14 \pm 2.248$	$0.76 \pm 0.041$
1, 2, 7, 8	1100 0011	$69.66 \pm 1.177$	$67.87 \pm 1.233$	$71.97 \pm 1.723$	$0.65 \pm 0.032$
1, 2, 3, 4	1111 0000	$70.60 \pm 1.426$	$68.00 \pm 1.521$	$73.95 \pm 1.945$	$0.64 \pm 0.036$
5, 6, 7, 8	0000 1111	$70.28 \pm 1.922$	$71.32 \pm 2.096$	$68.94 \pm 2.387$	$0.63 \pm 0.044$
1, 4, 5, 6	1001 1100	$70.68 \pm 1.023$	$70.82 \pm 1.054$	$70.50 \pm 1.586$	$0.61 \pm 0.030$
1, 3, 4, 5, 6	1011 1100	$73.53 \pm 0.853$	$73.37 \pm 0.857$	$73.74 \pm 1.434$	$0.56 \pm 0.027$
All	1111 1111	$71.17 \pm 1.245$	$71.79 \pm 1.311$	$70.37 \pm 1.783$	$0.61 \pm 0.033$

**Table 3:** Impact of different bandwidths on breast density classification for test data.

Bandwidths		Total accuracy (%)	LD accuracy (%)	HD accuracy (%)	BR (0–2)
1	1000 0000	$70.94 \pm 1.126$	$67.38 \pm 1.044$	$75.52 \pm 1.575$	$0.65 \pm 0.026$
2	0100 0000	$67.70 \pm 0.914$	$64.43 \pm 0.936$	$71.90 \pm 1.483$	$0.71 \pm 0.032$
3	0010 0000	$68.15 \pm 2.072$	$63.62 \pm 2.273$	$73.96 \pm 2.518$	$0.73 \pm 0.027$
4	0001 0000	$67.25 \pm 1.172$	$62.95 \pm 1.229$	$72.76 \pm 1.716$	$0.74 \pm 0.026$
5	0000 1000	$68.75 \pm 1.444$	$63.62 \pm 1.540$	$75.34 \pm 1.962$	$0.73 \pm 0.048$
6	0000 0100	$64.08 \pm 2.165$	$58.93 \pm 2.377$	$70.69 \pm 2.604$	$0.82 \pm 0.048$
7	0000 0010	$66.11 \pm 1.594$	$61.61 \pm 1.709$	$71.90 \pm 2.098$	$0.77 \pm 0.028$
8	0000 0001	$66.26 \pm 1.256$	$61.88 \pm 1.325$	$71.90 \pm 1.793$	$0.76 \pm 0.032$
1, 3, 5, 7	1010 1010	$68.98 \pm 0.817$	$66.71 \pm 0.821$	$71.90 \pm 1.399$	$0.67 \pm 0.026$
2, 4, 6, 8	0101 0101	$70.11 \pm 1.687$	$67.11 \pm 1.821$	$73.96 \pm 2.176$	$0.66 \pm 0.026$
3, 4, 5, 6	0011 1100	$69.73 \pm 1.997$	$67.52 \pm 2.189$	$72.59 \pm 2.450$	$0.65 \pm 0.048$
1, 2	1100 0000	$71.32 \pm 0.843$	$68.46 \pm 0.840$	$75.00 \pm 1.429$	$0.63 \pm 0.025$
7, 8	0000 0011	$64.83 \pm 1.816$	$60.67 \pm 1.965$	$70.17 \pm 2.298$	$0.79 \pm 0.026$
1, 2, 7, 8	1100 0011	$70.42 \pm 1.169$	$67.79 \pm 1.225$	$73.79 \pm 1.715$	$0.65 \pm 0.027$
1, 2, 3, 4	1111 0000	$70.04 \pm 1.452$	$66.44 \pm 1.548$	$74.65 \pm 1.971$	$0.67 \pm 0.026$
5, 6, 7, 8	0000 1111	$70.57 \pm 1.882$	$72.08 \pm 2.056$	$68.62 \pm 2.347$	$0.63 \pm 0.047$
1, 4, 5, 6	1001 1100	$71.70 \pm 1.009$	$71.54 \pm 1.041$	$71.90 \pm 1.572$	$0.58 \pm 0.026$
1, 3, 4, 5, 6	1011 1100	$73.66 \pm 0.855$	$74.50 \pm 0.859$	$72.59 \pm 1.436$	$0.57 \pm 0.029$
All	1111 1111	$71.17 \pm 1.212$	$72.21 \pm 1.279$	$69.83 \pm 1.751$	$0.60 \pm 0.026$

### Breast cancer detection

In this section, the main idea is to evaluate the impact of having prior-knowledge about density label on cancer detection. In this current investigation, we used the radiologist BD reference standard as prior knowledge about density label. In future work, our aim is increasing the accuracy of our BD model, using its own output for providing the prior knowledge about density (to be then used in cancer detection). In general, based on the available BD information, all training samples were divided into two main categories: HD and LD. Separate AI-based models were then developed to detect breast cancer within each group. During the testing stage, once the BD label of an unknown sample was identified, the corresponding density-specific model was applied to predict whether the breast is healthy or non-healthy.

The dataset used for this investigation is the same as described in Table 1, where 1,010 samples were labelled as healthy (including completely healthy and benign lesions) and 187 samples were labelled as non-healthy (tumour lesions). Same as BD classification, best features, suitable classifiers, and appropriate combination of bandwidths can play important roles to develop a ML or AI–based models to detect breast cancer. It is worth noting that the same methods have been considered for this section. Table 4 represents the top 3 models for detecting breast cancer on test data, providing individual reports of the sensitivity and specificity for low- and high-density breasts.

**Table 4:** Breast cancer detection without prior knowledge of breast density for test data.

Feature + classifier	Bandwidths	Low density		High density	
		Specificity (%)	Sensitivity (%)	Specificity (%)	Sensitivity (%)
FFT + NB	0011 1000	$50.48 \pm 1.819$	$64.45 \pm 1.819$	$60.04 \pm 2.023$	$47.73 \pm 1.727$
FFT + NB	0000 0111	$55.80 \pm 0.972$	$63.86 \pm 1.373$	$56.08 \pm 0.801$	$46.53 \pm 1.490$
SF + SVM_linear	0000 0001	$50.14 \pm 1.631$	$63.83 \pm 1.914$	$50.81 \pm 1.519$	$59.45 \pm 1.930$

On the other hand, if there is prior knowledge about BD, it is possible to develop different models for each class of density to detect breast cancer. Table 5 shows the sensitivity and specificity for each BD class.

**Table 5:** Breast cancer detection with prior knowledge about the density of the breast for test data.

Low density				High density			
Feature + classifier	Bandwidths	Specificity (%)	Sensitivity (%)	Feature + classifier	Bandwidths	Specificity (%)	Sensitivity (%)
SF + NB	0000 0001	$60.61 \pm 0.53$	$68.02 \pm 1.21$	FFT + NB	0001 0000	$75.96 \pm 0.78$	$59.45 \pm 1.39$
SF + NB	0100 0000	$65.13 \pm 0.61$	$60.91 \pm 1.819$	FFT + NB	0010 0000	$74.71 \pm 0.92$	$59.45 \pm 1.71$
FFT + NB	0011 1100	$62.32 \pm 0.81$	$64.25 \pm 1.45$	FFT + NB	0000 0100	$73.38 \pm 0.87$	$47.73 \pm 1.89$

## Discussion and conclusion

Breast density is a critical factor influencing both breast cancer risk and the efficacy of current screening approaches. Dense fibroglandular tissues significantly increase breast cancer risk, elevating it four to six-fold compared to fatty breast tissue. Furthermore, dense breast tissues often mask tumours on mammographic images, resulting in increased interval cancer rates due to reduced sensitivity^10,11^. Recognizing these limitations, we leveraged MammoWave’ s inherent broadband sensing to develop a frequency-selective FFT analytical method that quantifies breast density directly from raw S21 scattering parameters. This technique operates in real-time, requires no image reconstruction, and eliminates exposure to ionizing radiation.

Our analytical framework centred on dual-domain FFT processing of raw S21 scattering signals acquired using MammoWave’ s rotational antenna setup. Spatial FFT across receiving antenna positions reveals large-scale scattering distributions, directly correlating with adipose or fibroglandular composition. Simultaneously, spectral FFT across the 1–9 GHz operating band isolates dielectric dispersion characteristics unique to tissue permittivity. For each frequency band, we computed magnitude and phase and statistical metrics such as the mean and standard deviation yielding both macroscopic and microscopic tissue heterogeneity.

As mentioned earlier, this study pursued two main objectives. The first was to develop a reliable and robust model for classifying BD groups, and the second was to investigate how prior knowledge of BD groups influences breast cancer detection. For the first objective, we conducted an ablation analysis beginning with the FFT. Since the FFT produces complex outputs with both magnitude and phase components, we also considered the number of FFT coefficients as a potential parameter. The results showed that combining phase and magnitude information while limiting the analysis to the first 40 FFT components improves classification performance. Physically, these low-order coefficients capture the dominant scattering behaviour of breast tissue, whereas higher-order terms mainly represent noise and minor oscillations. From a ML perspective, reducing the number of features to 40 helps minimize redundancy and overfitting while preserving the most discriminative information—explaining their effectiveness in BD classification.

To ensure that the proposed features and classifiers could produce an optimal BD model, we compared our FFT-based features with conventional ones commonly used in the literature. Although SF and those extracted using PCA yielded promising results, especially for identifying high-density breasts, achieving consistent performance across both density groups was crucial. The proposed FFT-based features demonstrated balanced and acceptable performance in this regard.

We also examined several classifiers to identify the most suitable approach for BD classification. Ensemble-based methods such as RF, AdaBoost, and XGBoost achieved near-perfect training accuracy due to their strong capacity to model complex nonlinear relationships by combining multiple weak learners. However, their performance on validation and test sets revealed mild overfitting. Among all models, SVM-RBF and RF achieved the best trade-off between accuracy and generalization, with approximately 70% total accuracy and relatively low BR values. This confirms their robustness in handling nonlinear feature boundaries derived from microwave frequency data.

In contrast, DL models such as DNN, LSTM, and 1D-CNN exhibited limited generalization and unstable convergence. This was mainly because such architectures typically require larger datasets and continuous feature representations, while our dataset consisted of a moderate number of samples and handcrafted frequency-domain features. Consequently, classical ML methods—particularly SVM-RBF and tree-based ensembles—proved more effective for the nature and scale of this problem. Based on the overall performance and BR rate, SVM-RBF showed the best results. The final step in developing the optimal BD model involved identifying which combinations of frequency bands provide the highest performance. The physical rationale behind the selected bands is based on the complementary propagation behaviours of low and high frequencies. Lower frequencies (around 1 GHz) penetrate dense breast tissue more effectively, characterizing the overall tissue permittivity with minimal attenuation. In contrast, higher frequencies (above 4 GHz) capture fine tissue structures associated with glandular microarchitecture, offering higher spatial resolution^1,2^.

As presented in Tables 2 and 3 for the test and validation data, the five optimal frequency bands (1, 3, 4, 5, and 6 GHz) were selected from the broader spectrum. This selection enhances the relevance of the extracted features while reducing dimensional complexity, ensuring computational efficiency suitable for point-of-care implementation. Specifically, sub-band 1 (around 1 GHz) provides deep tissue penetration and represents the global dielectric properties of the breast, while the mid-range bands (3–6 GHz) strike a balance between penetration and resolution—making them sensitive to tissue composition and heterogeneity between low- and high-density cases. Very high frequencies (above 7 GHz) are more prone to attenuation and noise, and very low frequencies offer less discriminative power. Therefore, combining low- and mid-frequency sub-bands yields the most informative features for classification.

Applying the SVM-RBF classifier to these handcrafted frequency-domain features resulted in an overall accuracy of 73.66% and a BR of 0.57 when distinguishing between low- and high-density breast tissues. Visualization of the decision boundaries in the optimized feature space clearly demonstrated distinct class separability, emphasizing SVM’s capability to effectively utilize frequency-specific metrics for differentiating tissue classes based on their permittivity characteristics. This work represents the first standalone use of microwave scattering analytics for BD classification, extending MammoWave’s functionality beyond lesion detection and dielectric contrast imaging. Beyond diagnostic classification, the quantitative density metrics derived from MammoWave can support risk-adapted screening strategies—where patients with high-density profiles may be directed toward higher-sensitivity imaging modalities, while those with low-density profiles can safely continue with standard mammographic screening. Notably, incorporating BD classification results into microwave-based cancer detection significantly enhanced diagnostic performance. Previously, classifiers without density information demonstrated limited sensitivity (approximately 47–64%) and modest specificity (50–60%). However, density-informed classifiers markedly improved these metrics, achieving sensitivity and specificity of 59.4% and 75.9% respectively for high-density cases, and 68.0% sensitivity with 60.6% specificity for low-density scenarios. This substantial improvement underscores the importance of integrating BD awareness into lesion-detection imaging algorithms, offering a density?informed thresholding mechanism, potentially enhancing detection sensitivity in challenging dense-tissue contexts and reducing false positive rates.

Looking forward, the synergy between MammoWave-derived density measures and automated lesion-detection algorithms promises a unified, density?informed workflow. Embedding real-time density profiles into detection models can dynamically adjust signal-processing parameters to account for tissue-specific scattering differences, further improving sensitivity in dense breast tissue. Moreover, longitudinal density mapping across patient cohorts will enable personalized risk modelling, informing bespoke screening intervals and preventive interventions.

From a clinical standpoint, our frequency?centric technique furnishes several transformative benefits: the absence of ionizing radiation permits safe, repeated density assessments, supporting longitudinal monitoring of density dynamics in high?risk cohorts and chemoprevention trials; real-time FFT outputs empower immediate clinical triage, allowing clinicians to refer HD patients to adjunct imaging (ultrasound or contrast?enhanced MRI) within the same encounter, thus mitigating interval-cancer occurrences^8,9^; MammoWave’ s streamlined characteristics could enable its rapid deployment in a wide array of settings from community health clinics and mobile screening units to resource-limited environments, thereby broadening access to advanced, non-ionizing BD assessment. By incorporating BD labels into ML pipelines trained on MWI data, we aim to evaluate the extent to which anatomical variability affects microwave signal propagation and tumour detection. This research not only explores the potential of MWI in dense breast but also establishes a radiation-free, data-driven framework for personalized breast cancer screening. Our findings have the potential to reshape current screening practices, offering a viable alternative or complement to mammography particularly for women with dense breast tissue who are often underserved by conventional methods.

## Data Availability

Data are available from the corresponding author, Mehran Taghipour-Gorjikolaie (email: mehran.taghipour-gorjikolaie@lsbu.ac.uk), upon reasonable request.
